# Dynamic Environmental Conditions Affect the Composition of a Model Prebiotic Reaction Network

**DOI:** 10.1021/jacs.3c00908

**Published:** 2023-03-24

**Authors:** Peer van Duppen, Elena Daines, William E. Robinson, Wilhelm T. S. Huck

**Affiliations:** Institute for Molecules and Materials, Radboud University Nijmegen, Heyendaalseweg 135, 6525 AJ Nijmegen, The Netherlands

## Abstract

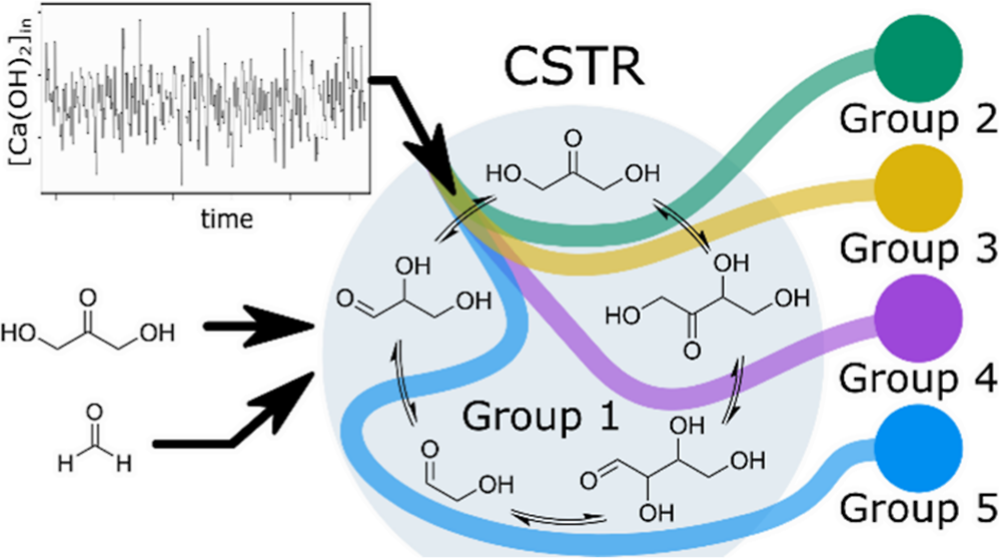

Prebiotic environments are dynamic, containing a range of periodic and aperiodic variations in reaction conditions. However, the impact of the temporal dynamics of environmental conditions upon prebiotic chemical reaction networks has not been investigated. Here, we demonstrate how the magnitude and rate of temporal fluctuations of the catalysts Ca^2+^ and hydroxide control the product distributions of the formose reaction. Surprisingly, the product compositions of the formose reaction under dynamic conditions deviate significantly from those under steady state conditions. We attribute these compositional changes to the non-uniform propagation of fluctuations through the network, thereby shaping reaction outcomes. An examination of temporal concentration patterns showed that collections of compounds responded collectively to perturbations, indicating that key gating reactions branching from the Breslow cycle may be important responsive features of the formose reaction. Our findings show how the compositions of prebiotic reaction networks were shaped by sequential environmental events, illustrating the necessity for considering the temporal traits of prebiotic environments that supported the origin of life.

## Introduction

Understanding how prebiotic chemical systems evolved toward primitive life remains a major scientific challenge. Many prebiotically plausible synthetic pathways to key building blocks of living systems have been reported,^1−6^ and together they provide an outline of the compositional landscape accessible from prebiotic precursors.^7^ During the evolutionary process toward life, these building blocks were incorporated in available prebiotic chemical reaction networks (CRNs) destined to form the various metabolic and replicative networks necessary for primitive life.^4,8−13^ However, the driving forces and mechanisms behind this evolution are unknown. It has been postulated that the structure and behavior of prebiotic CRNs originating in prebiotic waters were shaped by environmental traits that constrained their reaction outcomes toward those with a propensity to support living systems.^8−11^ Such conditions would have been dynamic, just as they are on modern Earth, varying on a variety of timescales such as the diurnal cycle and precipitation patterns.

Due to constant conditional variations, a significant part of the chemistry embedded in the prebiotic environment must have been out of equilibrium, a key characteristic of living systems. Thus, understanding the evolution of prebiotic chemistry toward life requires not only the knowledge of possible chemical transformations given fixed environmental traits but also an understanding of how dynamic conditions shaped the structure and composition of prebiotic CRNs, as necessitated by their fate to create life. The environment on early Earth is often discussed in terms of static properties, for example, the oxidation state of the atmosphere^14^ or the pH of the ocean. Perhaps due to the nature of evidence available, reports on the dynamics of Archaean Earth consider fluctuations on geological timescales,^15−17^ but calculations have been devised to model chemical weathering rates.^18^ Prebiotic chemistry investigations have followed suite, and reactions are typically performed in a series of well-defined steps at constant pH, temperature, or mineral composition which purport to mimic the prebiotic environment.^1−3,6,9−11,19,20^ Other investigations have considered wet–dry or dry-down dynamics on the outcomes of prebiotic reactions, which provide concentration and dehydration mechanisms.^19,21,22^ However, the dynamic conditional aspects of prebiotic Earth, such as the diurnal cycle and precipitation patterns, may have imposed their temporal signatures on reaction networks embedded within the environment. In dynamic environments, the timescales of individual reactions may vary significantly in comparison to one another and relative to the external dynamics, meaning that these reaction networks may not achieve a steady state. We lack concepts and model systems for studying such out-of-equilibrium reaction networks. Developing a “dynamic” view of prebiotic chemistry will enable the exploration of reaction outcomes driven by environmental dynamics, revealing new avenues in the reaction and system design.

**Figure 1:**
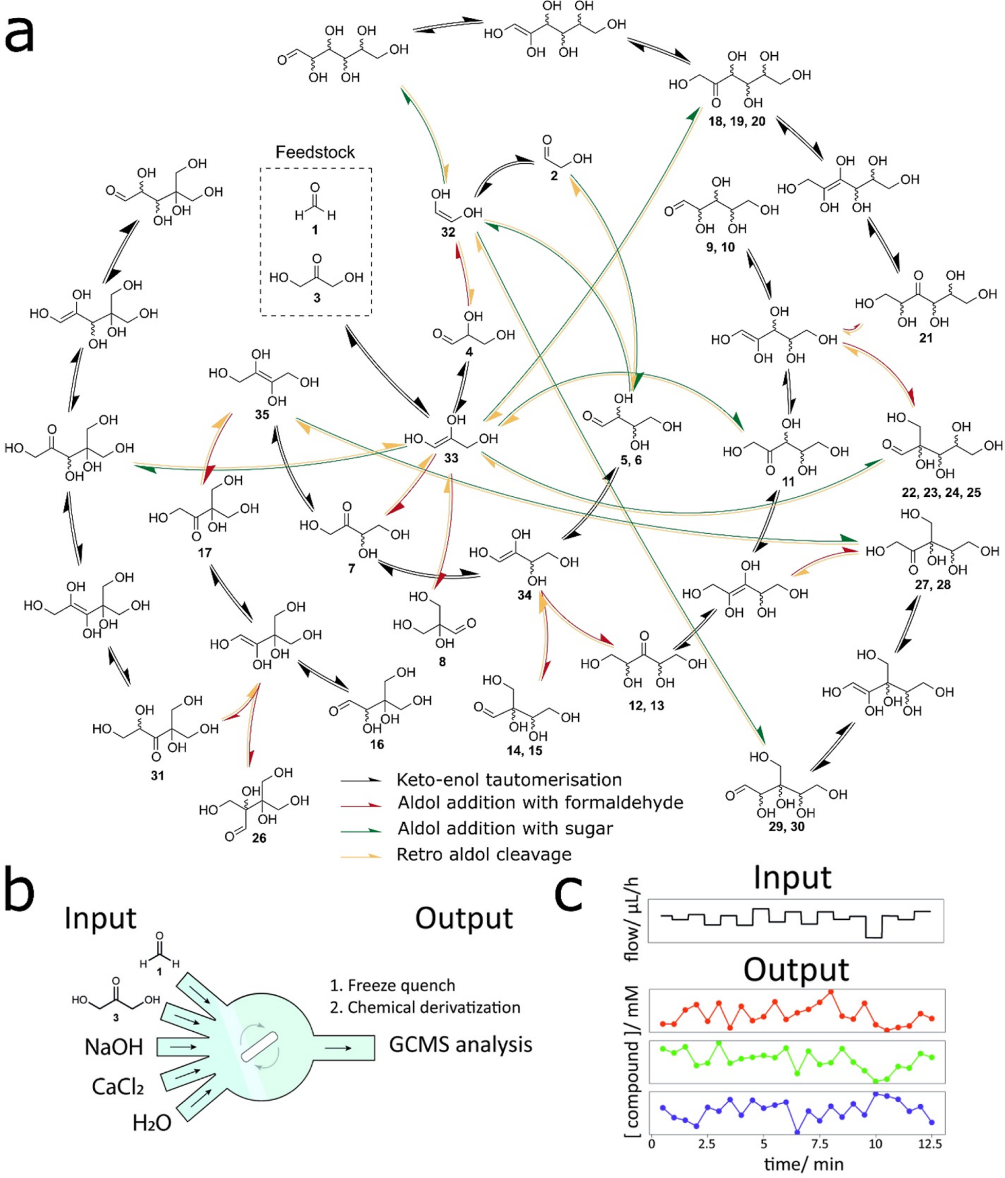
Formose reaction network and the experimental setup: (a) Schematic representation of the combinatorial explosion in the formose reaction; Cannizzaro reactions are omitted. (b) Schematic representation of a CSTR used to perform the formose reaction under out-of-equilibrium conditions. Input flow rates of CaCl_2_ and NaOH were modulated over time and balanced with the inflow of water to maintain a constant residence time (see Figure S2 for a more detailed scheme of the flow setup). (c) Examples of an input for Ca^2+^/hydroxide concentration profile and output carbohydrate concentration profiles. For each experimental condition, 50 datapoints were collected, in which the concentrations of 28 compounds were determined from GC–MS chromatograms.

Understanding how environmental dynamics are translated into the behavior of CRNs is a vital first step in understanding how prebiotic systems adapted to their environment. We have recently shown that product compositions of the formose reaction, a model prebiotic reaction system, are the result of inherent interactions between chemical reactivity and the environment. Using simple, prebiotically plausible feedstocks such as formaldehyde and glycolaldehyde, it creates compositional patterns consisting of monosaccharides in response to varying steady-state environmental conditions.^23−28^ These reactions can be catalyzed using Earth-abundant catalysts, for example, Ca(OH)_2_. The reactivity of the formose reaction can be rationalized using enolate, aldol addition, and Cannizzaro reactions, providing a well-defined space from which underlying reaction pathways can be inferred (Figure 1a).^23−27,29,30^ Thus, the formose reaction is an excellent model system which provides access to a variety of reactions, compounds, and conditional sensitivities under “one pot” conditions.

Here, we aim to elucidate the effect of time-dependent input variables on the composition and reaction connectivity of abiotic chemical reactions. To this end, we explored the effect of continually varying fluctuations in the concentrations of Ca^2+^ and hydroxide on the formose reaction. By varying the magnitude and the rate of change in these dynamics, we find that they propagate non-uniformly through the formose reaction and direct its compositional outcomes. These results demonstrate how dynamic conditions, as opposed to a fixed average condition, may have directly influenced the composition of prebiotic CRNs. Furthermore, we propose a structural basis for this behavior. Groups of compounds respond in unison to dynamic inputs. This grouping arises from varying sensitivities of specific branches of the formose reaction which radiate from a central Breslow cycle.^29,30^ The presence of these “subnetworks” indicates a striking similarity to biochemical reaction pathways,^31^ thus hinting at how prebiotic CRNs could have evolved into the subsystems of life, guided by the environment.

## Experimental Section

### Materials

d-Threose, l-erythrose, l-erythrulose, d-xylulose, d-ribulose (aqueous solution), d-talose, l-idose (aqueous solution), l-gulose, d-allose, d-altrose, and 1,3-dihydroxyacetone were purchased form Carbosynth Ltd.; l-(−)-sorbose, d-tagatose, and d-psicose were purchased from TCI Europe. CaCl_2_, NaOH, dihydroxyacetone, ribose, paraformaldehyde, N,O-bis(trimethylsilyl)trifluoroacetamine, *O*-ethylhydroxylamine hydrochloride, and pyridine were purchased from Sigma-Aldrich. Formaldehyde solution was prepared by depolymerization of paraformaldehyde by heating in water at 60 °C. Ultrapure water (from a Veolia system) was used to prepare all aqueous solutions. All other chemicals were used without further purification.

### Instrumentation

Gas-chromatography–mass spectrometric analysis was performed on JEOL JMS-100GCv. The gas chromatograph Agilent 7890A GC was equipped with an HP-5MS column (length: 30 m, inner diameter: 0.25 mm, film thickness: 0.25 μm). The injector inlet was set to a temperature of 250 °C, and for sample analysis (injection volume: 1 μL), the split mode was applied (ratio: 1/10). The GC was used with the following temperature program: oven temp/°C: 100, 170, 210, 250, 325; rate/°C min^–1^: 0, 14, 4, 15, 60; time/min: 2.33, 0, 0, 0, 3.75. Helium was used as a carrier gas (flow rate: 1 mL/min). For the mass analysis, a JEOL AccuTOF mass spectrometer was used with an Electron Impact Ionization Mode.

## Experimental Section

A detailed discussion of parameters chosen in this study is included in the Supporting Information.

### Flow Reactions

Flow reactions were performed in a cone-shaped continuous stirred-tank reactor (CSTR, volume = 411 μL) with five inlet channels at the bottom and an outlet channel at the top (Figure S2). It was fabricated by polydimethylsiloxane according to a previously reported procedure.^32^ Five gastight Hamilton syringes (25 mL) were filled with solutions of formaldehyde (198 mM), DHA (198 mM), NaOH (240 mM), CaCl_2_ (120 mM), and water. The syringes were mounted on high precision Cetoni Nemesys syringe pumps and attached to the inlet channels of the CSTR. The syringe pumps were controlled with Nemesys software, in which a flow profile was loaded for each syringe.

Experimental conditions are given in Table 1. For each experimental condition, the CSTR was left to equilibrate to the applied dynamics for 30 min before 50 samples were collected with time intervals of 40.8 s (or 30.6 s for EXP013). These sampling intervals were selected to sample with a frequency matching or exceeding the rate of change in the dynamic flow input, given the drop rate (10.2 s) of the reactor outlet. Some samples were removed from the analysis due to spoilage during the derivatization process. Groups of experiments were performed on the same day with equilibration periods between them (EXP001-EXP003, EXP004-EXP006, EXP007-EXP009, and EXP010-EXP012). EXP013 was performed in a single run.

**Table 1:** Experimental Conditions^a^

experiment	σ_Ca(OH)2,in_/mM	step rate/s	[formaldehyde]_in_/mM
EXP001	0.00	0	50
EXP002	2.89	45	50
EXP003	5.75	45	50
EXP004	0.00	0	20
EXP005	2.89	45	20
EXP006	5.75	45	20
EXP007	0.00	0	100
EXP008	2.89	45	100
EXP009	5.75	45	100
EXP010	0.00	0	50
EXP011	5.75	120	50
EXP012	5.75	45	50
EXP013	5.75	30; 60; 120	50

a All experiments were performed with fixed inlet concentrations of formaldehyde ([formaldehyde]_in_) as indicated and dihydroxyacetone (50 mM). Inlet concentrations of CaCl_2_ were sampled from a Gaussian distribution with average 15 mM and standard deviations as indicated (σ_Ca(OH)_2_,in_) and applied via the modulation of the input flow rate of the CaCl_2_ solution with a frequency indicated by the step rate. The inlet concentration (flow rate) of NaOH was varied in unison with the inlet of CaCl_2_ to maintain a constant 1:2 ratio of Ca^2+^/HO^–^. The flow rates applied to EXP013 were created from a linear combination of concentrations selected from three independent Gaussian distributions (mean = 15 mM), which varied over three different timescales as indicated. The flow rate of a separate water inlet was varied to offset the varying flows of CaCl_2_ and NaOH to maintain a constant residence time (120 s). Temperature: 21 °C.

### GC–MS Derivatization

Samples from the CSTR outlet were derivatized according to previously reported protocols.^33−35^ Aliquots (35 ± 0.1 μL) were collected from the CSTR outlet, immediately flash-frozen in liquid nitrogen, then freeze-dried overnight to give white solids. The samples were randomized before proceeding with derivatization so that any random error introduced during the derivatization process was spread across the samples. *O*-ethylhydroxylamine in pyridine (75 μL, 20 mg/mL) was added to each sample. The samples were heated and shaken (70 °C, 30 min, 600 rpm), then left to cool. Bis(trimethylsilyl)trifluoroacetamide (25 μL) was added, and the samples were shaken and heated again (*T* = 70 °C, 30 min, 600 rpm). The samples were allowed to cool down to room temperature, and both dodecane and tetradecane in pyridine (100 μL, 1.6 mM) were added. The samples were then centrifuged (3 min, 13,000 rpm). The supernatant was decanted into analytical vials for analysis by GC–MS (see the Instrumentation section).

### GC–MS Data Processing

GC–MS peak integration and assignment were performed as previously reported on raw chromatographic data using a program written in the programming language Python with the Numpy (1.22.2)^36^ and SciPy (1.8.0)^37^ packages.^23^ Peak assignment was performed by comparison of peak retention times and mass spectra with reference chromatograms. Peaks which could not be assigned using reference chromatograms were assigned by inferring their identity based on the retention time, fragmentation pattern, and consideration of the assignment likelihood given the other compounds produced in the experiment (see Figure 4a). Quadratic calibration curves (see Table S1) were used to convert peak integrals to compound concentrations. For non-commercially available compounds (branched C_4_–C_6_), for which calibrations were unavailable, an average calibration curve for all calibrated sugars with similar molecular weights was used. For compounds with two peaks in the chromatogram, the largest peak was used in the data analysis.

### Data Analysis and Interpretation

All data analyses were performed using the Numpy^36^ (1.22.2) and SciPy^37^ (1.8.0) Python libraries which are available at https://github.com/huckgroup/Formose_2022.git.

### Hierarchical Clustering Analysis

The hierarchical clustering analysis was performed on all experimental conditions that were perturbed with Ca(OH)_2_ fluctuations (Figure 4b). For the cluster analysis of each experimental condition, pairwise distances based on the “correlation” metric were calculated between time traces using scipy.spatial.distance.pdist(*x*) (eq 1) to create a distance matrix.
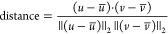
1where *u* and *v* are vectors of the concentration time profiles for compounds; *u̅* and *v̅* are the means of *u* and *v*, respectively; *x*·*y* is the dot product of *x* and *y*; and .

A hierarchical cluster analysis was performed on the *distance matrix*, by creating a *linkage matrix* with scipy.cluster.hierarchy.linkage (*distance matrix*), using the “*average*” method. At each step in the clustering, the nearest two clusters *A* and *B* were combined in a higher-level cluster *X*. The height of *X* in the dendrogram was calculated as δ(_*A*,*X*_) = δ_(*B*,*X*)_ = *d*_(*A*,*B*)_/2. The new distance matrix was calculated, where distances between newly formed cluster *A* ∪ *B* and cluster *Y* were calculated with eq 2.
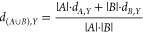
2where |*A*| and |*B*| are the number of elements in *A* and *B*. Thus, the newly calculated distance is a proportional average between distance *d*_*A*,*X*_ and *d*_*B*,*X*_. Clusters were assigned via the inspection of Figure 4b with reference to the underlying chemistry.

### Time-Interval Correlation Analysis

Correlation analyses were performed on discrete differentials at varying time intervals (30, 60, 90, 120, and 150 s) of concentration time traces of the Ca(OH)_2_ input against the measured outputs (compounds: **3**, **5**–**21**, **23**, **25**, **26**, **28**–**31**). First, the input of Ca(OH)_2_ was resampled by linear interpolation so that it had the same time axis as the output data. Sliding time windows (30, 60, 90, 120, and 150 s) were passed along the time progresses of the input and compound time–concentration profiles (Figure S14). The mean of the values within each time window was subtracted from the mean of the values in the previous window to create a vector of values indicating how each signal changes on average over the scale of the time window (*x*). One such vector was created for each time window width, for each input and compound time–concentration profile. This process is illustrated in Figure S14. The values in *x* were mean centered (*x* – np.mean (*x*)) and were normalized by dividing by the standard deviation with numpy.std (*x*) (eq 3).
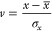
3where *v* is the mean-centered and scaled differential vector at a specific time interval, *x̅* is the mean of vector *x*, and σ_*x*_ is the standard deviation of *x*.

For each time window (30, 60, 90, 120, and 150 s), the Pearson correlation coefficient was calculated between the averaged and differentiated input (*v*_in_) and each of the corresponding output averaged differentials (*v*_out_) using scipy.stats.pearsonr (*v*_in_, *v*_out_) (eq 4).
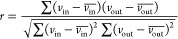
4where  and  represent the mean value of *v*_in_ and *v*_out_, respectively. A positive linear correlation is indicated by a Pearson correlation coefficient between 0 and 1, and an inverse linear correlation is indicated by a Pearson correlation coefficient between −1 and 0. A Pearson correlation coefficient of 0 indicates that there is no linear correlation between the input and the output.

## Results and Discussion

### Experimental Studies of the Formose Reaction in a Dynamic Environment

To provide an out-of-equilibrium system to which environmental dynamics may be applied, the formose reaction was performed in a CSTR (Figure 1b).^23^ This system allowed for time-resolved compositional measurements to be performed precisely by sampling from the outlet. The experimental conditions explored in this investigation are given in Table 1. Discretely varying input patterns of NaOH and CaCl_2_ were applied to the system by varying their input flow rates. Flow rates were varied to maintain a 1:2 ratio of Ca^2+^ to hydroxide. Thus, we refer to the applied environmental dynamics as the net fluctuation of Ca(OH)_2_. Flow rates were sampled from normal distributions around averages of [NaOH]_in_ = 30 mM and [CaCl_2_]_in_ = 15 mM with varying magnitudes (σ_[Ca(OH)_2_]in_ = 0.00, 2.89, and 5.75 mM). Two time intervals between changes in the flow rate were used (45 and 120 s). The flow rate of a syringe containing water was simultaneously adjusted against the flows of Ca^2+^ and HO^–^ solutions to maintain a constant residence time of 2 min.

The influence of temporal fluctuations on formose reactions was studied at a fixed inlet dihydroxyacetone concentration (50 mM) and three different inlet formaldehyde concentrations (20, 50, and 100 mM). These conditions covered previously identified organizational patterns which were dominated by either aldol addition reactions of C_2_ and C_3_ carbohydrates ([formaldehyde]_in_ = 20 mM)^23,24,28,38^ or by addition reactions of formaldehyde to sugar enolates in the network ([formaldehyde]_in_ = 100 mM)^23,25^ as well as a condition intermediate between the two ([formaldehyde]_in_ = 50 mM).^23^

For each experiment, 50 samples were collected from the output of the CSTR at 40.8 s intervals for EXP001–EXP009 and at 30.6 s intervals for EXP010–EXP013. Samples from the reactor outlet were freeze-quenched, lyophilized, and derivatized similarly to a previously reported protocol^23,33−35^ and analyzed by gas chromatography–mass spectrometry (GC–MS). Quantification was based on peak integrals of total ion chromatograms. Each peak was assigned to a compound via a combination of retention time and mass spectral fragmentation pattern (Figures S3–S5) and using authentic sample calibrations for linear chain sugars (Table S1). In total, 28 compounds were identified.

### Effect of the Magnitude in Variation of [Ca(OH)_2_] upon the Composition of the Formose Reaction

**Figure 2:**
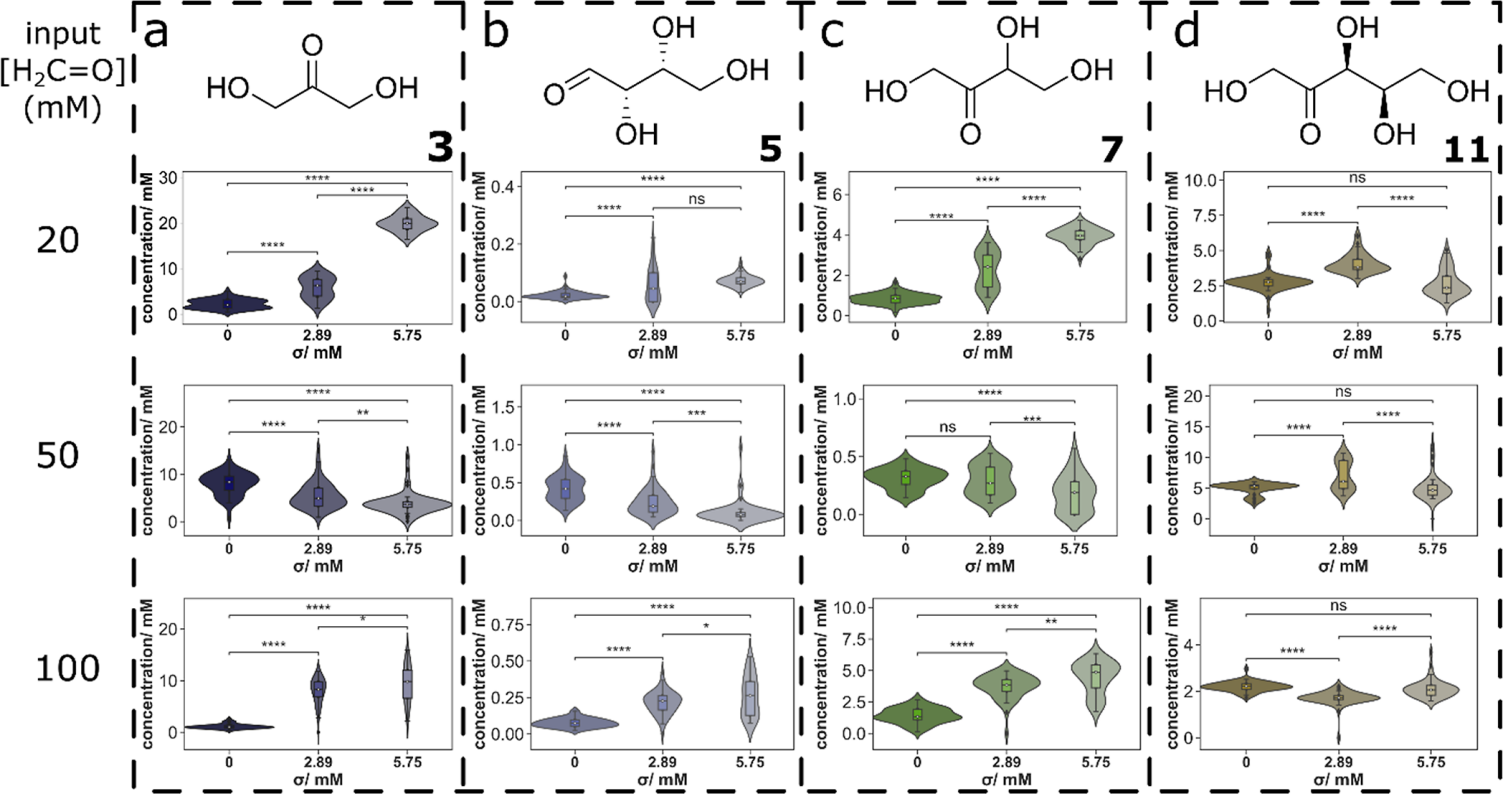
Concentration distributions of C_3_, C_4_, and C_5_ sugars in the formose reaction with varying Ca(OH)_2_ fluctuations. The distributions of (a) DHA (**3**), (b) threose (**5**), (c) erythrulose (**7**), and (d) xylulose (**11**) for varying concentrations of [formaldehyde]_in_ perturbed with [Ca(OH)_2_]_in_ of various amplitudes (σ = 0, 2.89, 5.75 mM). *p*-Values were calculated as indicators for the significance of the difference between means and are annotated as follows: ns (5 × 10^–2^ < *p* ≤ 1), * (1 × 10^–2^ < *p* ≤ 5 × 10^–2^), ** (1 × 10^–3^ < *p* ≤ 1 × 10^–2^), *** (1 × 10^–4^ < *p* ≤ 1 × 10^–3^), **** (*p* ≤ 1 × 10^–4^).

The effect of amplitude in the variation (σ) of [Ca(OH)_2_]_in_ on the concentrations of a number of formose products is demonstrated in Figure 2a–d (for all compounds, see Figures S6–S11). Surprisingly, despite the system having the same average concentrations of Ca(OH)_2_ compared to an unperturbed reaction, the measured concentration distributions vary significantly (at least *p* ≤ 1 × 10^–4^ in each series for compounds **3**, **5**, **7**, and **11**) in response to fluctuations in [Ca(OH)_2_]_in_. In the absence of applied input dynamics, some compounds (for example, **3**, **5**, and **7**, Figure 2a–c, respectively; 50 mM formaldehyde) exhibit a relatively broader distribution of responses compared to others (for example, **11**, Figure 2d). This variation is due to experimental noise introduced by factors such as the time taken for sample freeze-quenching and variations in mixing inside the reactor which we were unable to eliminate. This variance is constant across the data set.

The applied perturbations alter not only the average concentration of each compound but also the distribution and thus range of concentrations that can be accessed by each compound. Furthermore, the sensitivity and trends in response to formose reaction product concentrations to the applied dynamics are dependent on the formaldehyde concentration. A range of responses are observed across the conditions investigated (Figures S6–S11), hinting at the nonlinear behavior of the underlying formose reaction pathways. Figure 2 depicts four case studies which illustrate the breadth of this behavior. At formaldehyde concentrations of 20 mM, dihydroxyacetone (**3**, Figure 2a) and erythrulose (**7**, Figure 2c) exhibit significant increases in the average concentration in response to the increasing σ. On the other hand, the distribution of threose (**5**, Figure 2b) only responds significantly to increasing σ from 0.0 to 2.89 mM but not between 2.89 and 5.75 mM. Xylulose (**11**, Figure 2d) responds significantly to σ = 2.98 mM, but its concentration distributions are similar between σ = 0.00 and 5.75 mM. Increasing the formaldehyde concentration to 50 mM lowers the significance in the changes induced by increasing σ for **3** and **7**, while **5** becomes more responsive to higher magnitudes of input dynamics. **11**’s responses remain relatively similar to those observed with 20 mM formaldehyde. Further increasing the concentration of formaldehyde to 100 mM again results in little effect on the response of **11** to varying σ. The responses of **3** and **5** become more similar than those at lower concentrations of formaldehyde. Significant changes in their mean values are observed when dynamics are applied, but little change in their means is seen on increasing σ to 5.75 mM due to their high variance. **7** regains its sensitivity which was absent at 50 mM formaldehyde albeit with lower sensitivity than when the concentration of formaldehyde is 20 mM.

### Rate of Variation in [Ca(OH)_2_] Affects the Composition of the Formose Reaction

**Figure 3:**
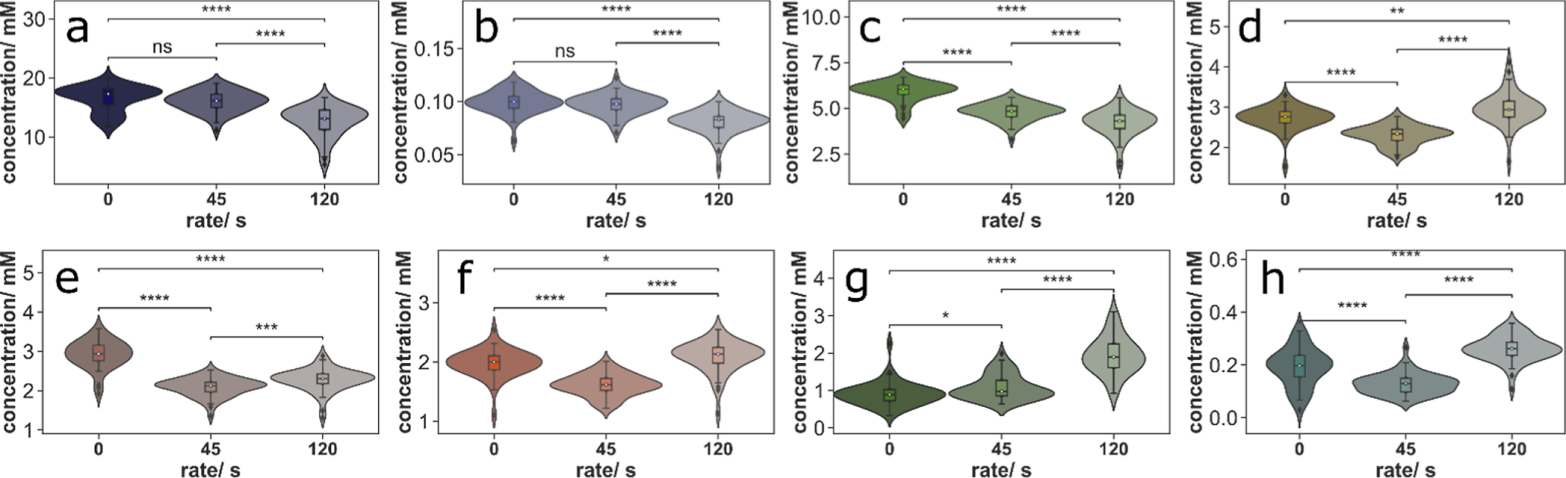
Distributions of compounds (a) **3**, (b) **5**, (c) **7**, (d) **11**, (e) **13**, (f) **15**, (g) **23**, and (h) **25** for the [formaldehyde]_in_ = 50 mM network at a steady state and perturbed with a 45 s or 120 s rate of change ([Ca(OH)_2_]_in_ σ = 5.75 mM). *p*-Values were calculated as indicators for the significance of the difference between means and are annotated as follows: ns (5 × 10^–2^ < *p* ≤ 1), * (1 × 10^–2^ < *p* ≤ 5 × 10^–2^), ** (1 × 10^–3^ < *p* ≤ 1 × 10^–2^), *** (1 × 10^–4^ < *p* ≤ 1 × 10^–3^), **** (*p* ≤ 1 × 10^–4^).

As fluctuations in the environment can occur at multiple timescales (for example, the terrestrial diurnal or tidal cycles can be relatively slower than precipitation or wind), we explored the response of the formose reaction to varying perturbations frequencies. Here, we compared the compositional change of the formose reaction in the presence of 50 mM formaldehyde at a steady state to that when exposed to varying rates of Ca(OH)_2_ input fluctuations (45 and 120 s, Figure 3) at a fixed average input concentration and magnitude of variation (σ). Again, we observed average concentrations of compounds which deviate from steady-state averages (**3**, **5**, **7**, and **11**; *p* ≤ 1 × 10^–4^, in all compound series; see Figures S12 and S13). Varying the rate of environmental change also has marked effects on the concentration distributions of formose products (Figure 3). The concentration of erythrulose (**7**) has the same standard deviation but lower average than when the input of Ca(OH)_2_ is varied at 45 s ([**7**] = 4.8 ± 0.5 mM) intervals than at a steady state ([**7**] = 6.0 ± 0.5 mM). However, when the frequency of variation is 120 s, the average concentration of **7** is lower relative to that at 45 s variation albeit with a higher standard deviation (4.2 ± 0.7 mM).

These compositional trends show how the formose reaction is sensitive to the rate of fluctuations in Ca(OH)_2_ input concentrations, leading to significant, but non-uniform, changes in concentrations of reaction products.

### Hierarchical Clustering and Correlation Analysis Identify Collective Responses

**Figure 4:**
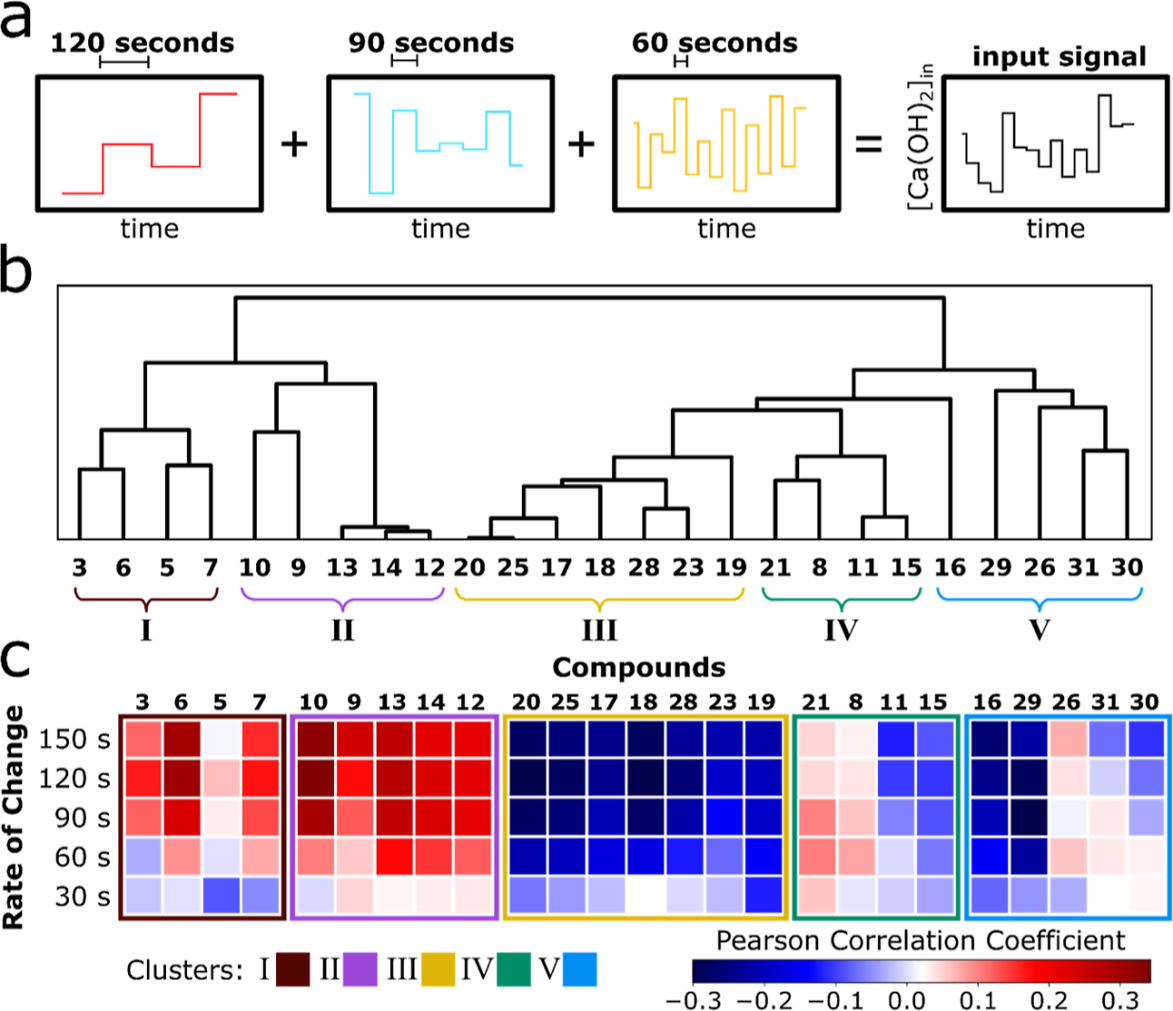
Superimposition of three timescales of variation in the Ca(OH)_2_ input (EXP013) reveal collective responses of compounds to conditional dynamics in the formose reaction. (a) An input consisting of variations on the scales of 60, 90, and 120 s was designed via the superimposition of three components. (b) Hierarchical clustering analysis of the concentration–time traces of EXP013 reveals partitioning into five groups of compounds (**I**–**V**) as indicated. (c) Correlation of each compound trace to input dynamics on five timescales. For each compound in this network, the time-interval correlation of the input and output differential was calculated over the concentration difference at different timescales in [Ca(OH)_2_]_in_ (150, 120, 90, 60, and 30 s).

The grouped responses of compounds (for example, compounds **3**, **5**, and **7** in Figure 2) observed in response to the Ca(OH)_2_ dynamics indicate that the formose reaction network may behave as a collection of subnetworks. To explore the transfer of environmental fluctuations through the network in greater depth, we performed an experiment in which the formose reaction was perturbed using a superimposition of input dynamics. A dynamic input was constructed from the combination of three signals made of values selected at random from normal distributions (μ_[Ca(OH)_2_]in_ = 15 mM, σ_[Ca(OH)_2_]in_ = 5.75 mM) which varied with three different frequencies (30, 60, and 120 s). The concentration of formaldehyde was 50 mM (Figure 4a).

We performed a hierarchical clustering analysis on the time–concentration traces from this experiment. The cluster analysis revealed the partitioning of the formose reaction products into five (**I**–**V**) clusters (Figure 4b). Within each cluster, compounds responded to the changes in the environment in a similar way. We also performed time-averaged-correlation analyses to uncover which embedded timescales in the input were transferred to the various compounds. Clear differences in correlation to the input at varying timescales were observed between the clusters (Figure 4c; see Figure S14 for a detailed description of the time-interval averaging analysis). The correlations were superimposed on the five clusters identified (**I**–**V**, Figure 4c). Positive (negative) correlations indicate that changes in concentrations of products move in the same (opposite) direction as the input fluctuation.

The compounds in cluster **I** (**3**, **5**, **6**, and **7**) have a strong positive correlation to the input signal on longer (150 s) input timescales (Figure 4c). Conversely, an increasingly weaker correlation is seen for the shorter time scales. Cluster **II** exhibits a similar trend, with even stronger positive correlations to the longer input timescales. A strong negative correlation to the input is seen for cluster **III** at all but the shortest timescales (30 s). Compounds in clusters **IV** and **V** show broadly a weaker overall correlation with the input dynamics in comparison to the other clusters. Compounds **16** and **29** are exceptions to this trend, especially on longer timescales. Though the identified reaction modules explain the clustering results well, they do not provide a complete description of the entirety of the complex behavior of the formose reaction.

### Transfer of Environmental Dynamics to Compounds Is Governed by the Structure of the Formose Reaction Network

**Figure 5:**
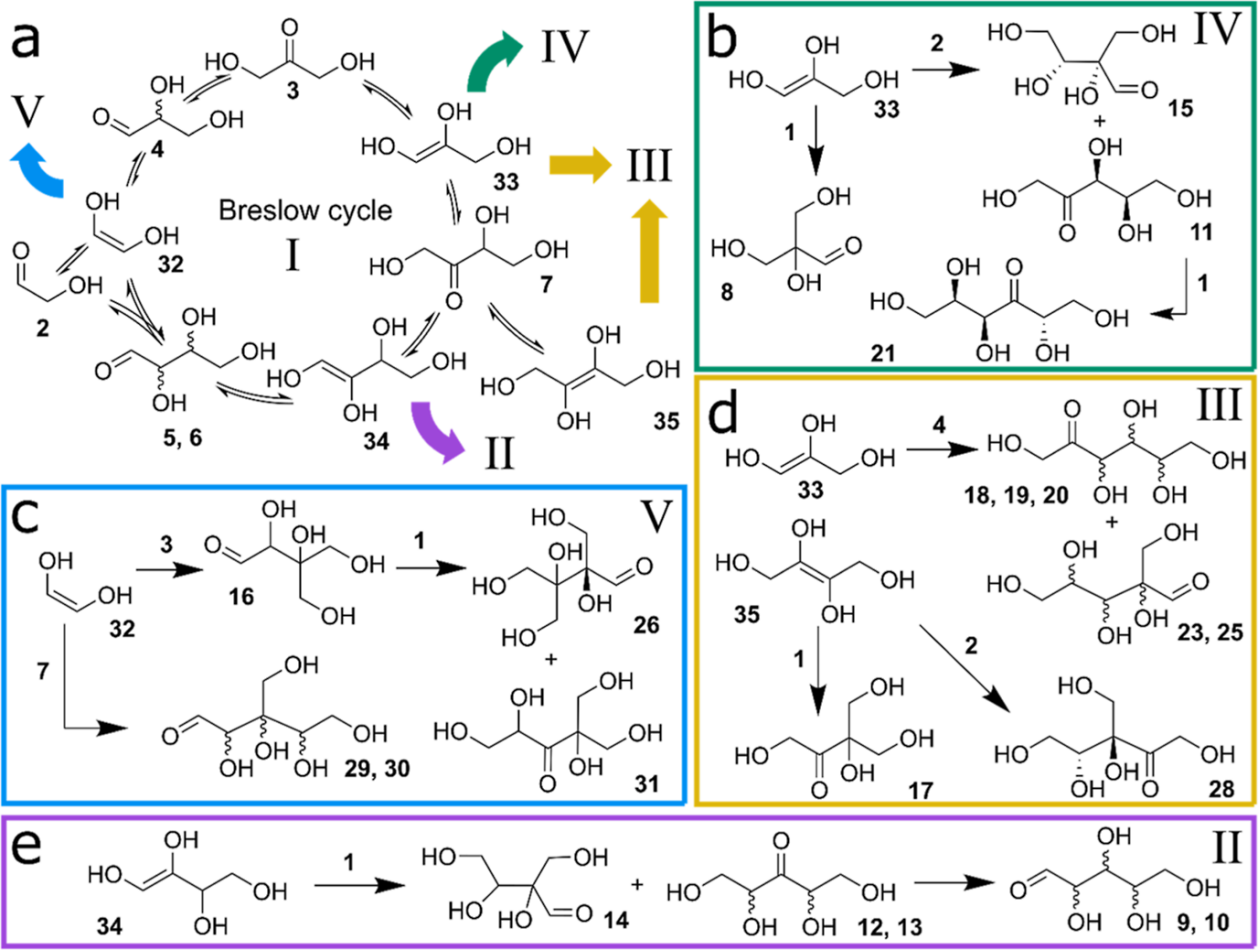
Identified clusters can be rationalized with gating reactions stemming from the central Breslow cycle. (a) 50 mM formaldehyde network, perturbed with multiple embedded rates (30, 90, and 120 s), represented around the core Breslow cycle. The gating reactions are indicated with colored arrows. (b–e) Gating reactions to produce the products in the respective cluster (**II**–**V**). Intermediate enolates are omitted for clarity.

A closer inspection of the compounds in the clusters identified in Figure 4b reveals that compounds typically associated with the Breslow cycle^29,30^ (**3**, **5**, **6**, and **7**, Figure 5a) remain within cluster **I** across all the variations in input conditions described above. These compounds also show a strong positive correlation with the input fluctuations and are also most sensitive to environmental perturbations, having higher standard deviations with respect to their average concentration compared to compounds in other clusters (Figures S6–S13).

Key to understanding the dynamics in the other clusters observed in the data are four enolate species (C_2_-enolate **32**, C_3_-enolate **33**, C_4_-1,2-enolate **34**, and the off-cycle C_4_-2,3-enolate **35**). These enolates control the gating reactions that connect the central cycle to the identified clusters of compounds (**II**–**V**) (Figure 4a).

The C_4_-1,2-enolate **34** is involved in reactions which gate access to cluster **II**, via aldol addition reactions with formaldehyde (Figure 5e). The products in cluster **III** form in addition reactions which involve the C_3_-enolate **33** and the off-cycle C_4_-enolate **35** (Figure 5d). Access to cluster **IV**, with xylulose (**11**) production, occurs via reaction with the C_3_-enolate **33** with glycolaldehyde (**2**) (Figure 5b). Cluster **V** is connected to the central cycle via aldol addition reactions between C_2_-enolate **32** and dihydroxyacetone (**3**) or erythrulose (**7**) (Figure 5c).

The varying connectivity of the clusters to the central cycle also explains the difference in the correlation strength for the different clusters. Gating reactions have varying degrees of sensitivity to [Ca(OH)_2_] fluctuations. As a result, they mediate the transfer of environmental dynamics to varying degrees to the compounds created downstream from them, while the Breslow cycle behaves as a central hub for the propagation of environmental fluctuations. Reactions with higher sensitivities allow for the transfer of faster conditional fluctuations compared to those with lower sensitivities, which only allow the propagation of fluctuations on longer timescales.

## Conclusions

In summary, we have shown how fluctuations in the environment are translated into product distributions via dynamic propagation through the formose reaction network. Rather than each compound responding uniquely to applied dynamics, collections of compounds respond together due to the structure of the underlying reaction network. Interestingly, we were able to attribute this structure to four “modules” of the formose reaction, which connect to a central Breslow cycle via as many gating reactions. These gating reactions control the propagation of environmental fluctuations through the network, thus producing the grouped behavior observed.

The adaptation of reaction networks to fluctuations in the environment has important implications for the field of prebiotic chemistry.^39,40^ Dynamic conditions must be considered in modeling prebiotic chemical processes and offer a mechanism through which complex chemical reactions may inherit information from the environment. The temporal signatures of reaction conditions offer an extra level of control on top of their average magnitudes in guiding the reaction outcomes of prebiotic reaction networks. Multiple reaction outcomes may be accessed from a single set of precursors, dependent on the rate and magnitude of variation of each parameter affecting the reaction. Time-dependent conditional variations may thus be used as a tool in controlling the compositions of prebiotic chemical reactions, for example, in guiding reactions toward the production of important building blocks such as ribose. Our results and interpretation demonstrate that there is a mechanistic basis to the compositional changes induced by dynamic environmental conditions. Reaction modularity offers a design principle upon which future investigations may be conceived in using dynamics to target specific collections of reactions and compounds. Thus, future experimental designs can be based on the promotion or inhibition reaction pathways in a mechanistic manner. Such investigations will provide a stronger conceptual union between the chemical properties of CRNs and environmental dynamics, thus unveiling mechanistic pathways to the evolution of life’s first biochemical processes.

Our results also underline that the consideration of environmental dynamics is essential in establishing scenarios for origins of life. It may be that the simple presence of a condition is not sufficient to give rise to biological systems. Specific regimes of time-dependent conditional variations may be required for life to evolve from prebiotic environments. Furthermore, the modular response of the formose reaction to environmental dynamics hints at how the modularity of extant biochemical pathways may have arisen in the absence of genetic inheritance or enzymatic catalysis, as has been proposed to have been the mechanism for the evolution of the core carbon metabolism.^4,5,41−43^

## Supporting Information Available

Discussion of the choice of reaction parameters in this study, schematic of the flow reactor setup, mass spectra of the assigned compounds, violin plots for all compounds, illustrations of analysis methods, and calibration factors (PDF)
Experimental data and Python scripts used to analyze and plot the data are available on GitHub (https://github.com/huckgroup/Formose_2022.git).

## Notes

Data and code supporting the findings of this study are available in the Supporting Information and on GitHub https://github.com/huckgroup/Formose_2022.git.
