# Supplementary material for: Dynamic Environmental Conditions Affect the Composition of a Model Prebiotic Reaction Network
Source: J Am Chem Soc. 2023 Mar 24;145(13):7559–68. doi: 10.1021/jacs.3c00908 (PMC10080678; doi:10.1021/jacs.3c00908)
Supplement: Supplementary file 1 — ja3c00908_si_001.pdf [file ja3c00908_si_001.pdf]

Supplementary Information

Dynamic Environmental Conditions Affect the Composition of a Model Prebiotic Reaction Network

Peer van Duppen[1], Elena Daines[1], William E. Robinson[1,*] and Wilhelm T. S. Huck[1,*]

[1] Institute for Molecules and Materials, Radboud University Nijmegen, Heyendaalseweg 135, 6525 AJ Nijmegen, The Netherlands

* Corresponding author. Email: w.huck@science.ru.nl, william.robinson@ru.nl

Table of Contents

Supplementary Discussion 2

Discussion of parameters chosen in this study 2

Selection of reaction conditions 2

Selection of $Ca(OH)_2$ amplitude series 2

Selection of multiple frequency input signal 2

Supplementary figures 4

Supplementary tables....................13

References 14

Supplementary Discussion

Discussion of parameters chosen in this study

Selection of reaction conditions

Previous work has shown that the inlet concentration of formaldehyde controls a compositional transition between 0 – 100 mM with $[\text{dihydroxyacetone}]_{in}$ = 50 mM, $[NaOH]_{in}$ = 30 mM and $[CaCl_2]_{in}$ = 15 mM.[1] We selected average input concentrations of formaldehyde, dihydroxyacetone, NaOH and $CaCl_2$ from within this space of parameters. Thus, the chosen conditional ranges provide access to a range of compositional outcomes.

Selection of $Ca(OH)_2$ amplitude series

Dynamic inputs of $[Ca(OH)_2]$ were generated by sampling from normal distributions (μ = 15 mM, σ = 2.89 mM or 5.75 mM) generated using Numpy[2] (1.22.2.) with function numpy.random.normal(). For the amplitude series $[NaOH]_{in}$ and $[CaCl_2]_{in}$ were varied around the steady state average (15 mM for $CaCl_2$ and 30 mM for NaOH) with varying amplitude randomly sampled from a normal distribution, (σ_{in} = 2.89 mM or 5.75 mM) of concentrations, varied via syringe flow rates as described in the Experimental Methods section Main Text. The magnitudes of variation were chosen such that they would perturb the reaction mainly via the applied dynamics, rather than via a bulk change in concentration. Flow rates where switched every 45 seconds, thus not allowing the catalyst or reaction to reach steady state to maintain a constant compositional variation. Note that NaOH and $CaCl_2$ syringes were not allowed to produce negative flow rates.

Selection of multiple frequency input signal

To construct the input signal containing variations on three timescales, the range 30 -120s was chosen. The residence time of the experiment was chosen as an upper bound (120 s),

with a lower bound of 30s to ensure that the applied dynamics could be sampled on a similar timescale.

Supplementary figures

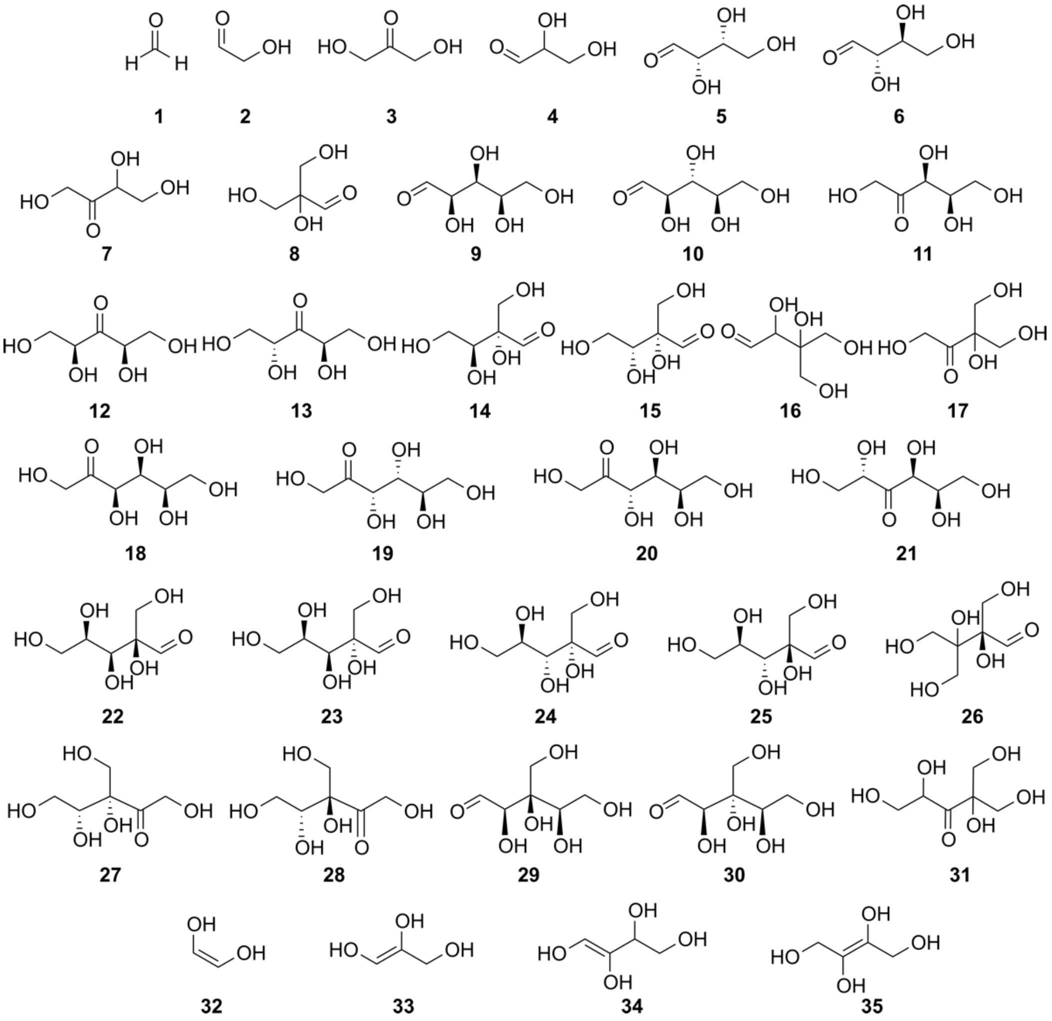

Figure S1. Structures of the compounds discussed in this work, with the corresponding numbering scheme (**1** – **35**).

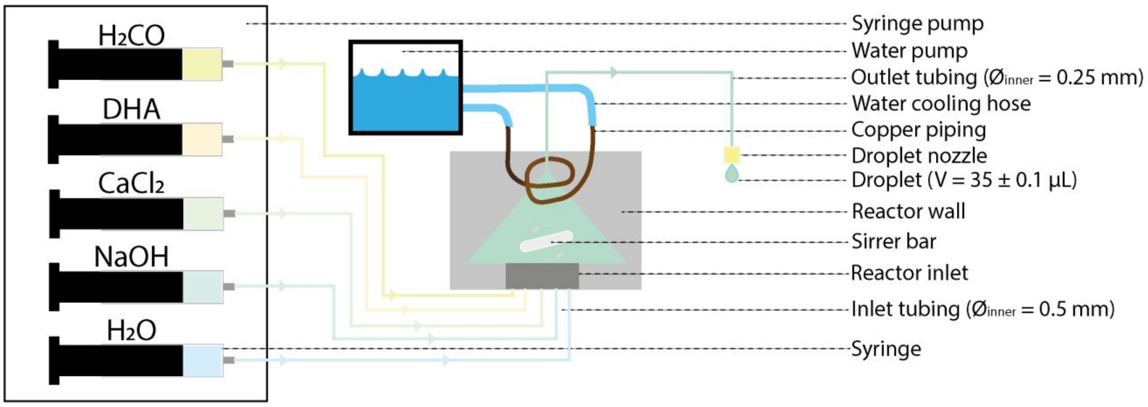

Figure S2. A detailed schematic representation of the flow reactor setup. The scheme indicates how five inlet syringes were connected to the bottom of a reactor constructed from polydimethylsiloxane (side view). The cone shaped reactor was temperature controlled and had the outlet at the top, which was connected to a droplet nozzle.

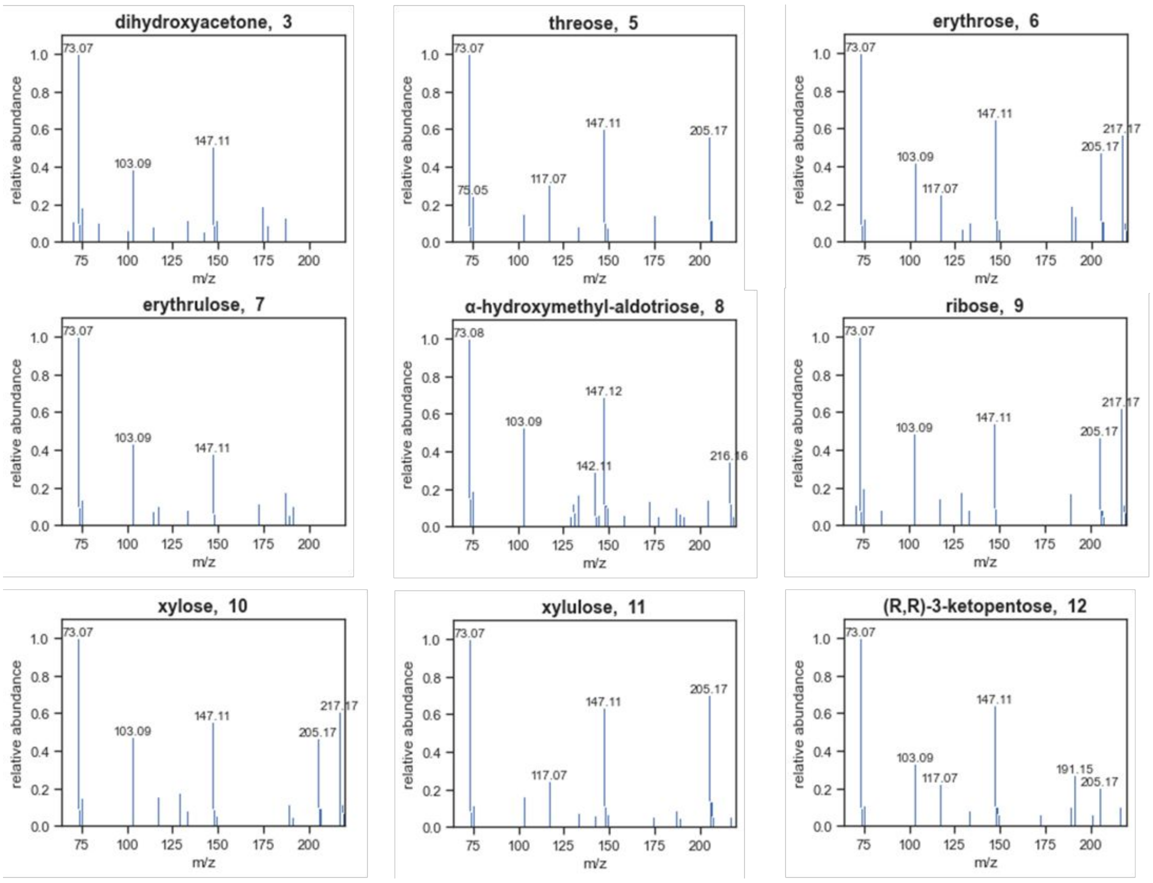

Figure S3. Mass spectra from GC-MS analysis of DHA (**3**), threose (**5**), erythrose (**6**), erythrulose, (**7**), α-hydroxymethyl-aldotriose (**8**), ribose (**9**), xylose (**10**), xylulose (**11**) and (R,R)-3-ketopentose (**12**).

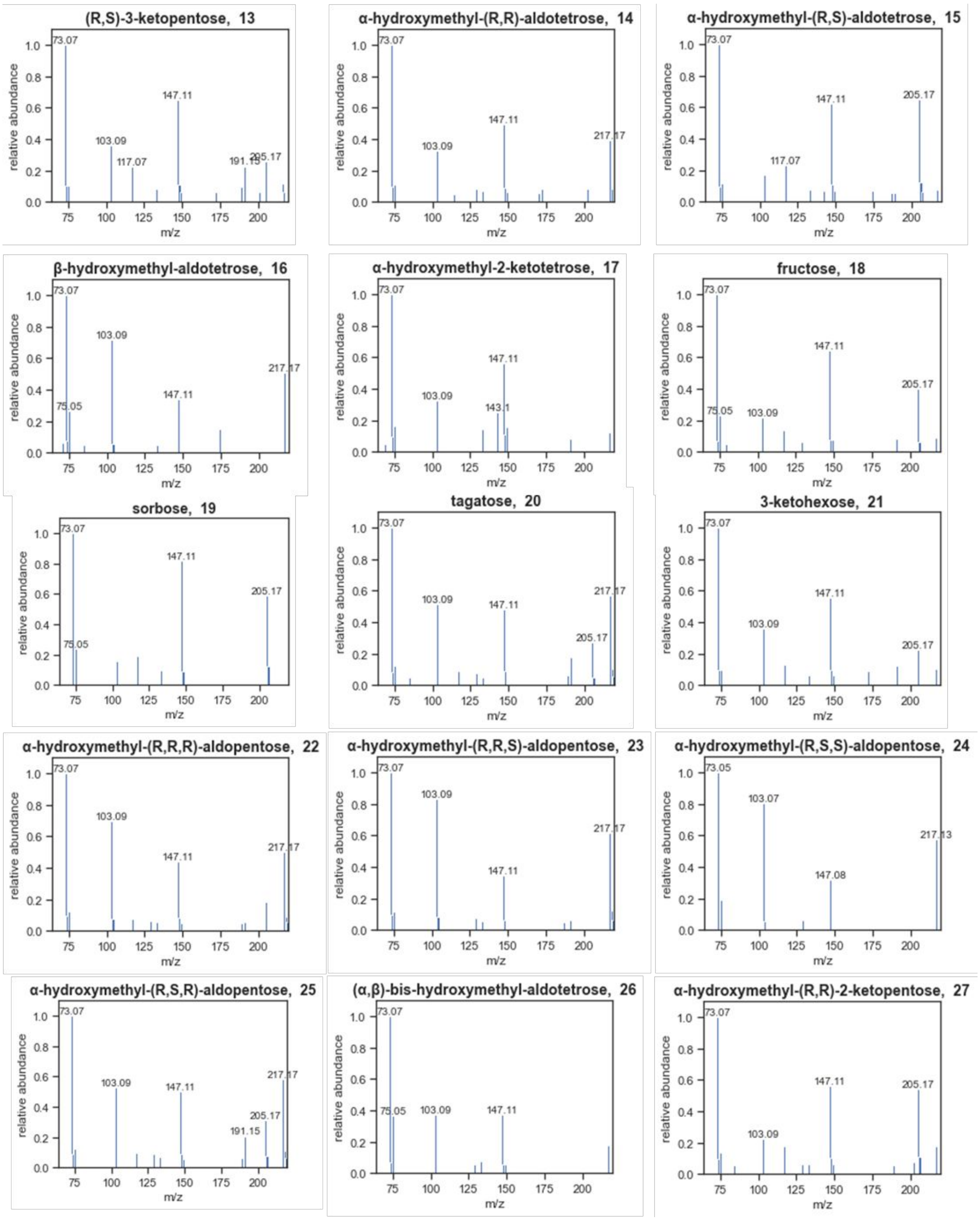

Figure S4. Mass spectra from GC-MS analysis for (R,S)-3-ketopentose (**13**), α-hydroxymethyl-(R,R)-aldotetrose (**14**), α-hydroxymethyl-(R,S)-aldotetrose (**15**), β-hydroxymethyl-aldotetrose (**16**), α-hydroxymethyl-2-ketotetrose (**17**), fructose (**18**), sorbose (**19**), tagatose (**20**), 3-ketohexose (**21**), α-hydroxymethyl-(R,R,R)-aldopentose (**22**), α-hydroxymethyl-(R,R,S)-aldopentose (**23**), α-hydroxymethyl-(R,S,S)-aldopentose (**24**), α-hydroxymethyl-(R,S,R)-aldopentose (**25**), (α,β)-bis-hydroxymethyl-aldotetrose (**26**) and α-hydroxymethyl-(R,R)-2-ketopentose (**27**).

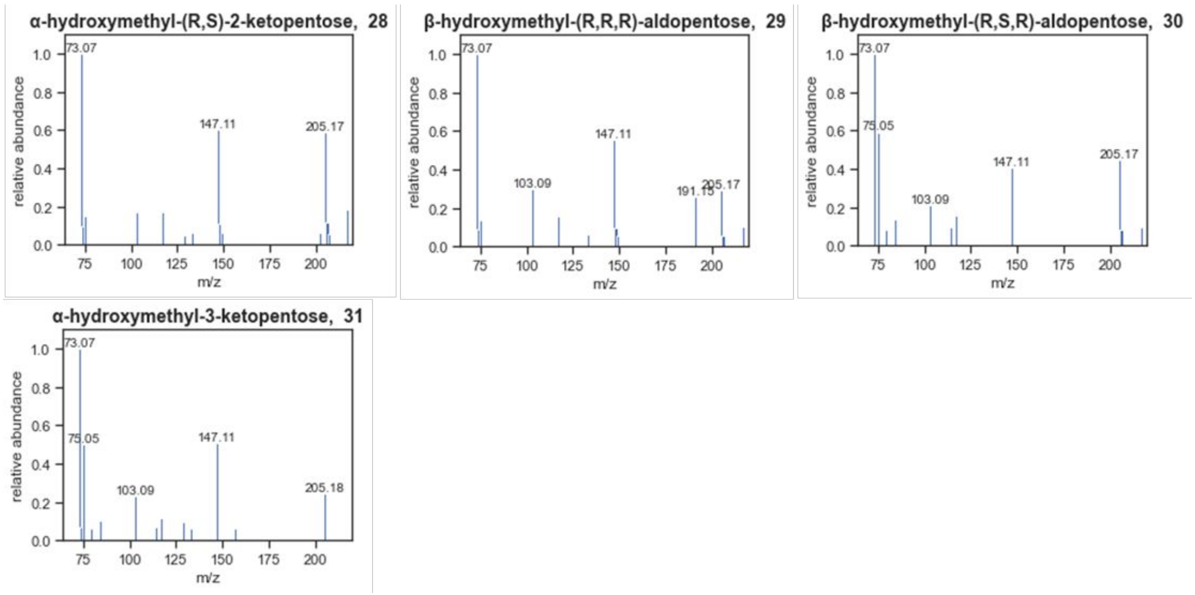

Figure S5. Mass spectra from GC-MS analysis for hydroxymethyl-(R,S)-2-ketopentose (**28**), β-hydroxymethyl-(R,R,R)-aldopentose (**29**), β-hydroxymethyl-(R,S,R)-aldopentose (**30**) and α-hydroxymethyl-3-ketopentose (**31**).

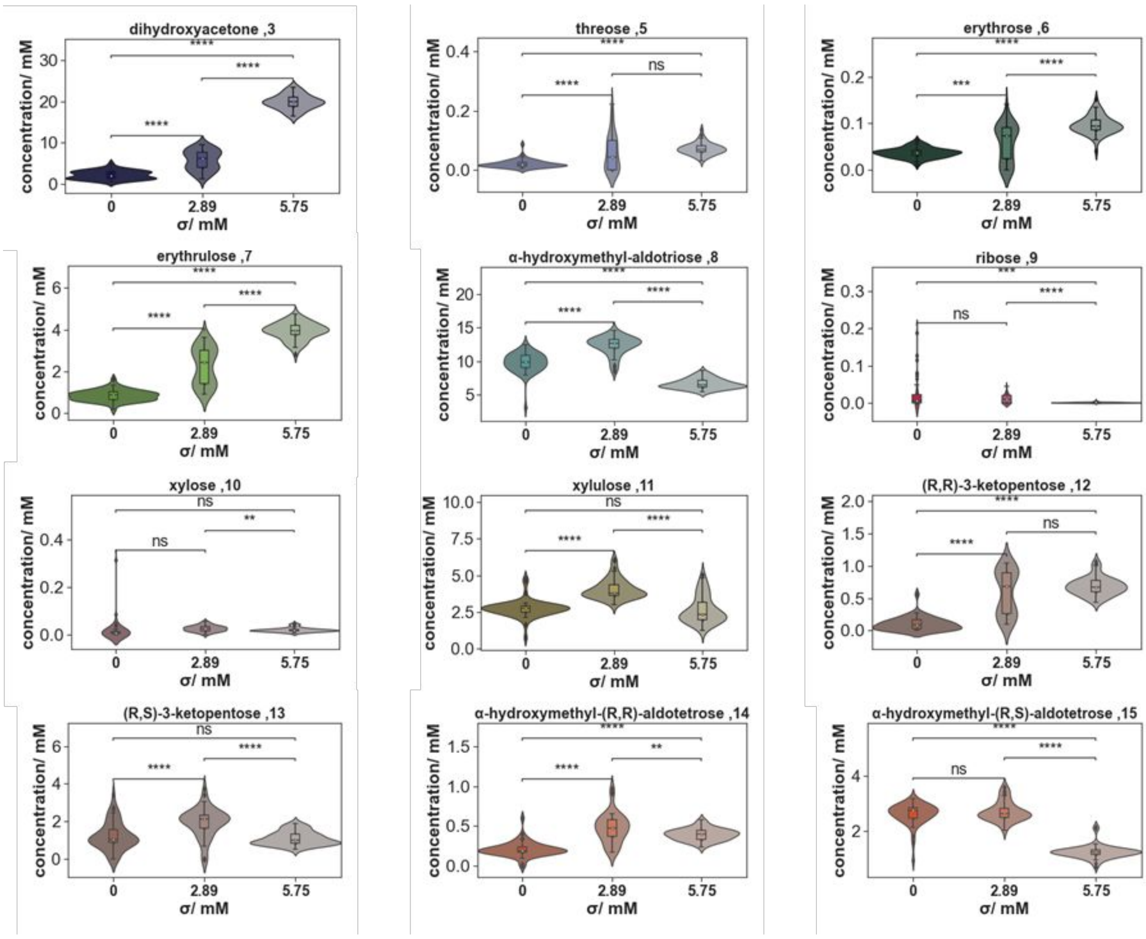

Figure S6. Violin plots of concentration distributions for identified compounds (**3**, **5**, **6**, **7**, **8**, **9**, **10**, **11**, **12**, **13**, **14**, **15**) at steady state, low amplitude and high amplitude ($\sigma_{[Ca(OH)2]in}$ = 0, 2.89 and 5.75 mM). Conditions: 20 mM $H_2C{=}O$, 50 mM DHA, 30 mM NaOH, 15 mM $CaCl_2$.

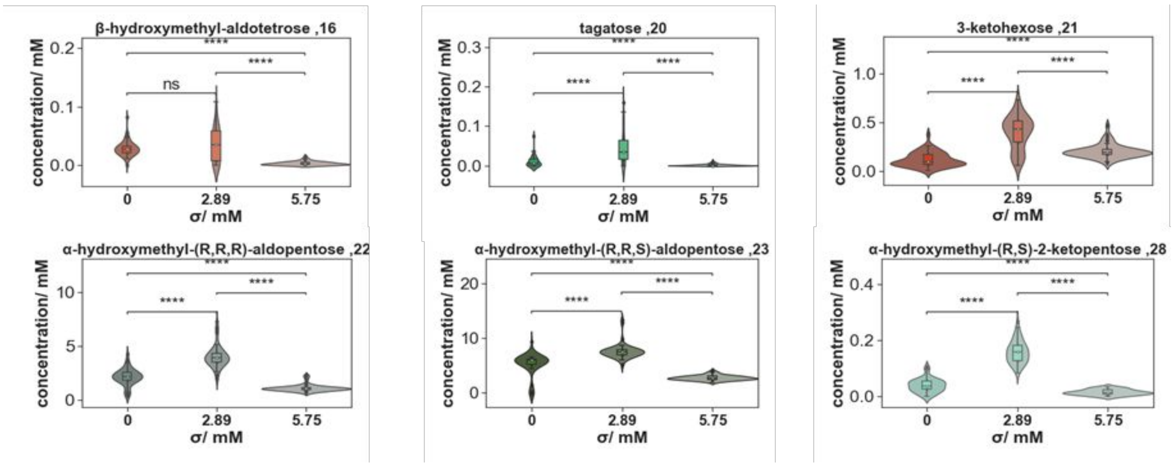

Figure S7. Violin plots for concentration profiles for the identified compounds (**16**, **20**, **21**, **22**, **23**, **28**) at steady state, low amplitude and high amplitude($\sigma_{[Ca(OH)2]in}$ = 0, 2.89 and 5.75 mM). Conditions: 20 mM $H_2C{=}O$, 50 mM DHA, 30 mM NaOH, 15 mM $CaCl_2$.

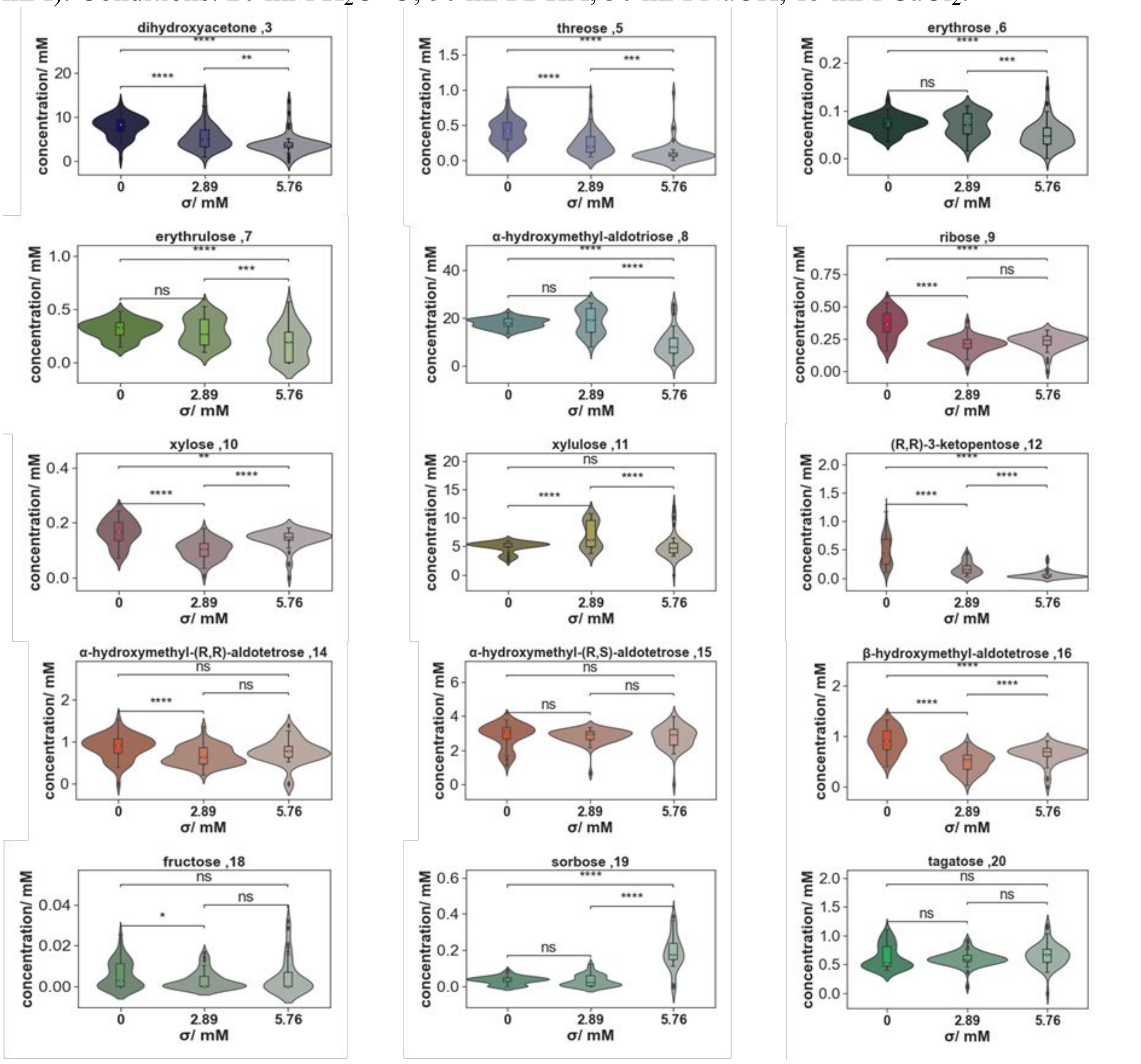

Figure S8. Violin plots of the concentration distributions for identified compounds (**3**, **5**, **6**, **7**, **8**, **9**, **10**, **11**, **12**, **14**, **15**, **16**, **18**, **19**, **20**) at steady state, low amplitude and high amplitude ($\sigma_{[Ca(OH)2]in}$ = 0, 2.89 and 5.75 mM). Conditions: 50 mM $H_2C{=}O$, 50 mM DHA, 30 mM NaOH, 15 mM $CaCl_2$.

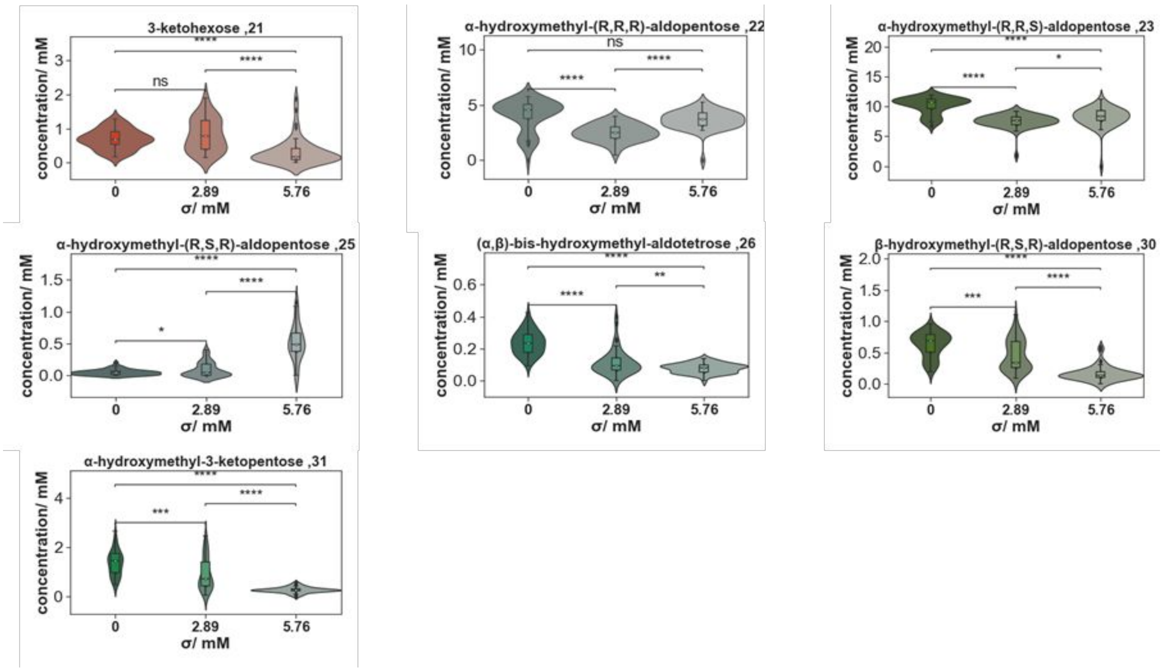

Figure S9. Violin plots for concentration distributions of the identified compounds (**21**, **22**, **23**, **25**, **26**, **30**, **31**) at steady state, low amplitude and high amplitude($\sigma_{[Ca(OH)2]in}$ = 0, 2.89 and 5.75 mM). Conditions: 50 mM $H_2C{=}O$, 50 mM DHA, 30 mM NaOH, 15 mM $CaCl_2$.

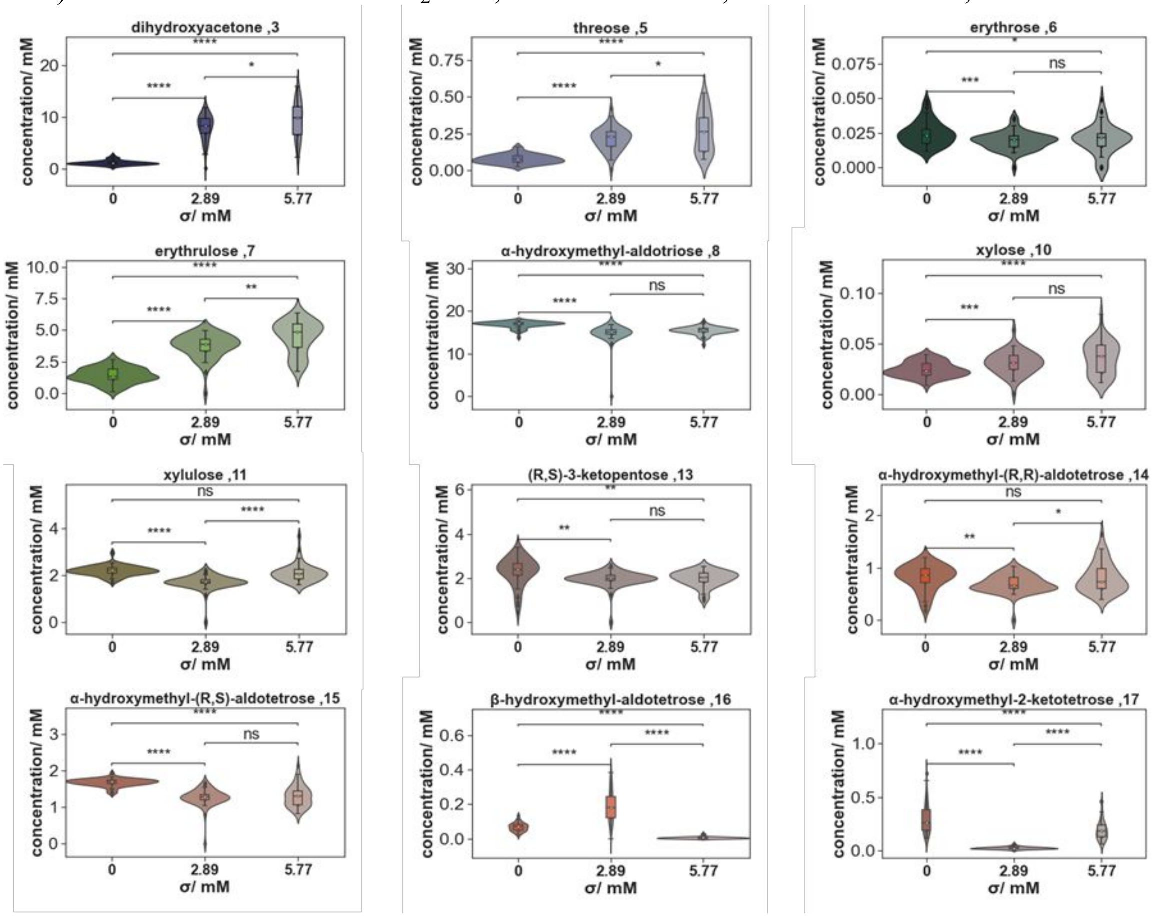

Figure S10. Violin plots for concentration distributions for the identified compounds (**3**, **5**, **6**, **7**, **8**, **10**, **11**, **13**, **14**, **15**, **16**, **17**) at steady state, low amplitude and high amplitude ($\sigma_{[Ca(OH)2]in}$ = 0, 2.89 and 5.75 mM). Conditions: 100 mM $H_2C{=}O$, 50 mM DHA, 30 mM NaOH, 15 mM $CaCl_2$.

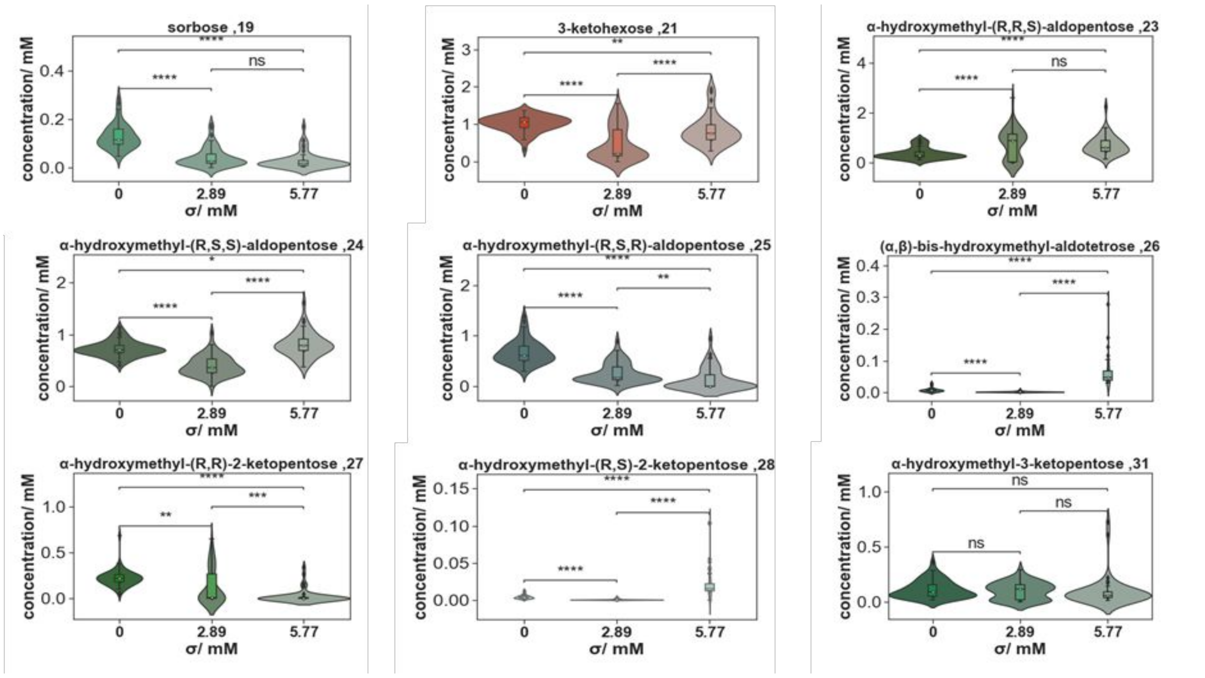

Figure S11. Violin plots for concentration distributions for the identified compounds (**19**, **21**, **23**, **24**, **25**, **26**, **27**, **28**, **31**) at steady state, low amplitude and high amplitude ($\sigma_{[Ca(OH)2]in}$ = 0, 2.89 and 5.75 mM). Conditions: 100 mM H_2C=O, 50 mM DHA, 30 mM NaOH, 15 mM $CaCl_2$.

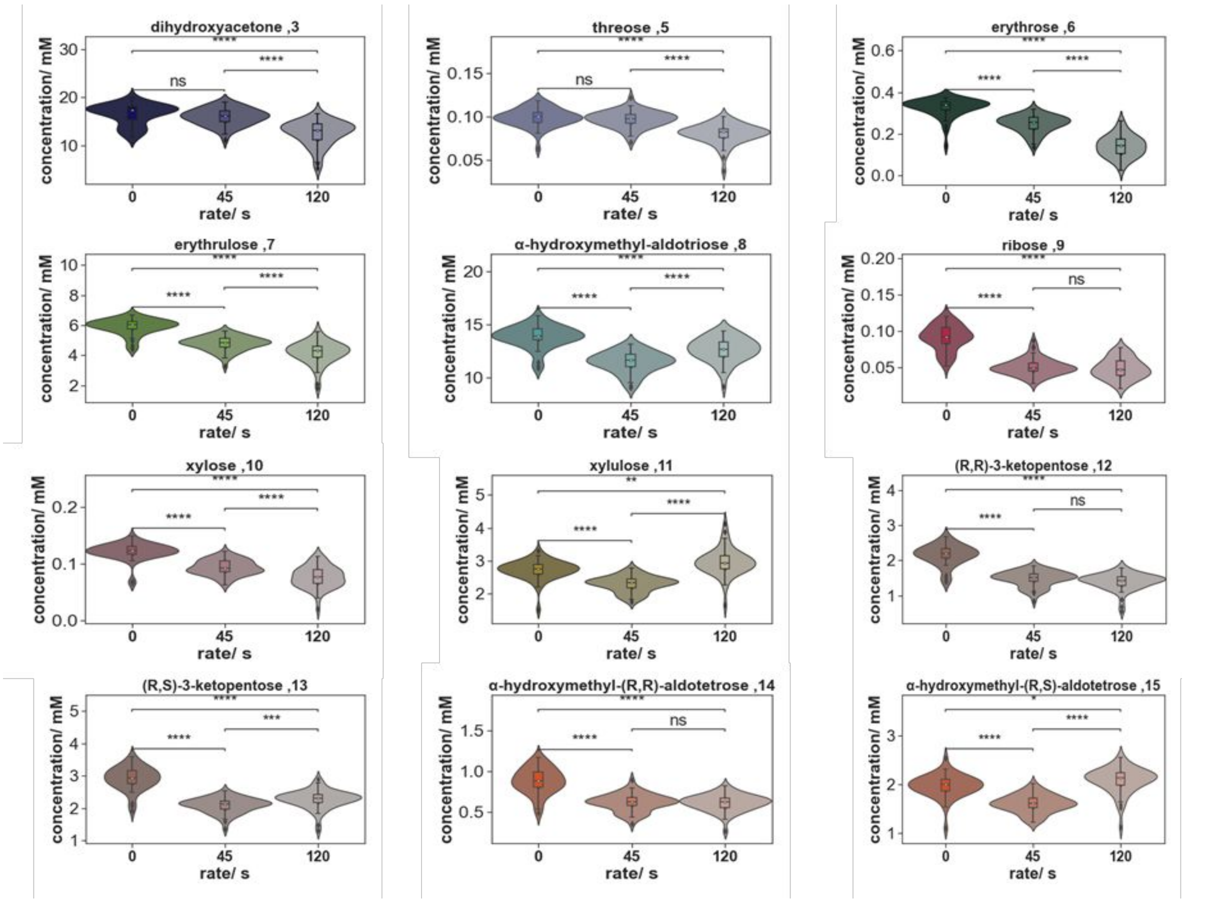

Figure S12. Violin plots for concentration distributions for the identified compounds (**3**, **5**, **6**, **7**, **8**, **9**, **10**, **11**, **12**, **13**, **14**, **15**) at steady state, 45 s and 120 s rate of change ($\sigma_{[Ca(OH)2]in}$ = 0, 5.75 and 5.75 mM). Conditions: 50 mM H_2C=O, 50 mM DHA, 30 mM NaOH, 15 mM $CaCl_2$.

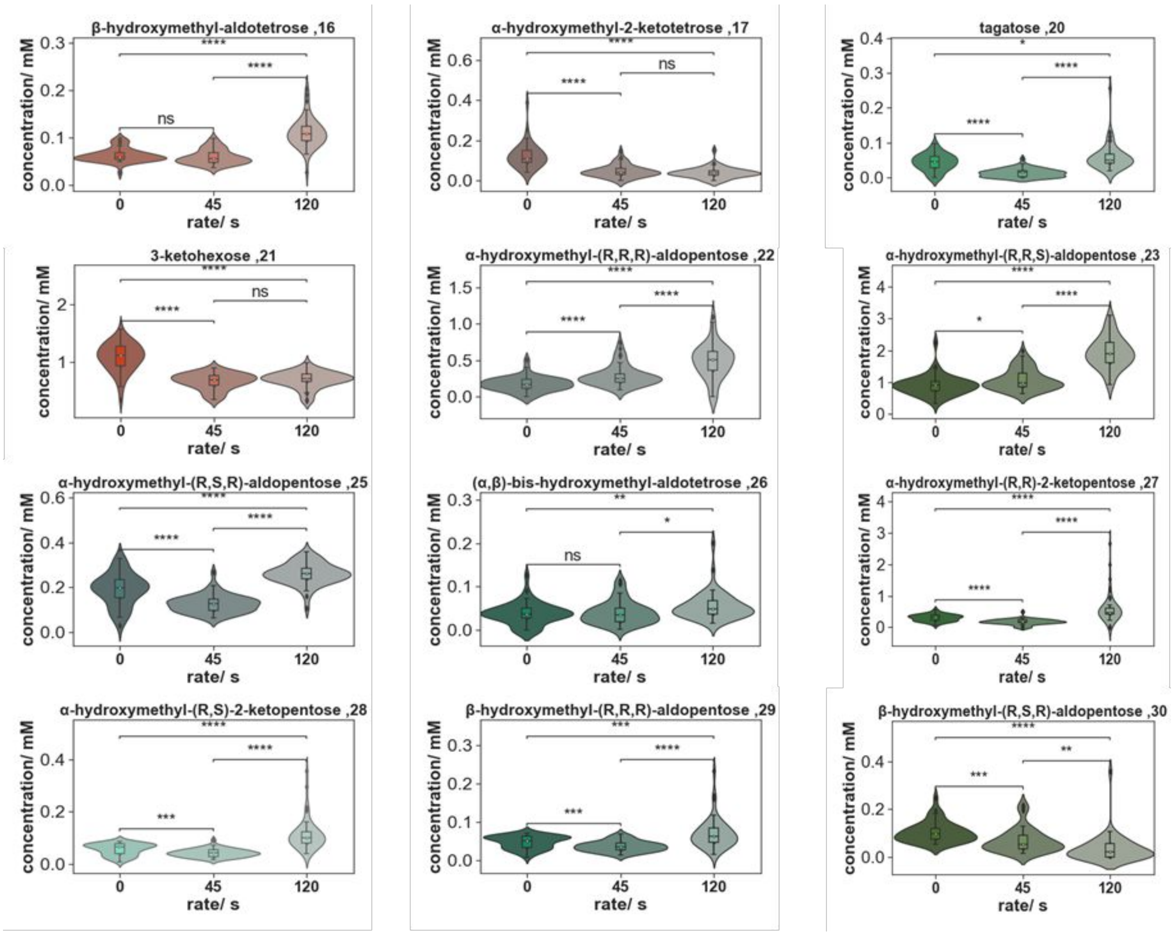

Figure S13. Violin plots for concentration distributions for the identified compounds (**16**, **17**, **20, 21**, **22**, **23**, **25**, **26**, **27**, **28**, **29**, **30**) at steady state, 45 s and 120 s rate of change ($\sigma_{[Ca(OH)2]in}$ = 0, 5.75 and 5.75 mM). Conditions: 50 mM $H_2C{=}O$, 50 mM DHA, 30 mM NaOH, 15 mM $CaCl_2$.

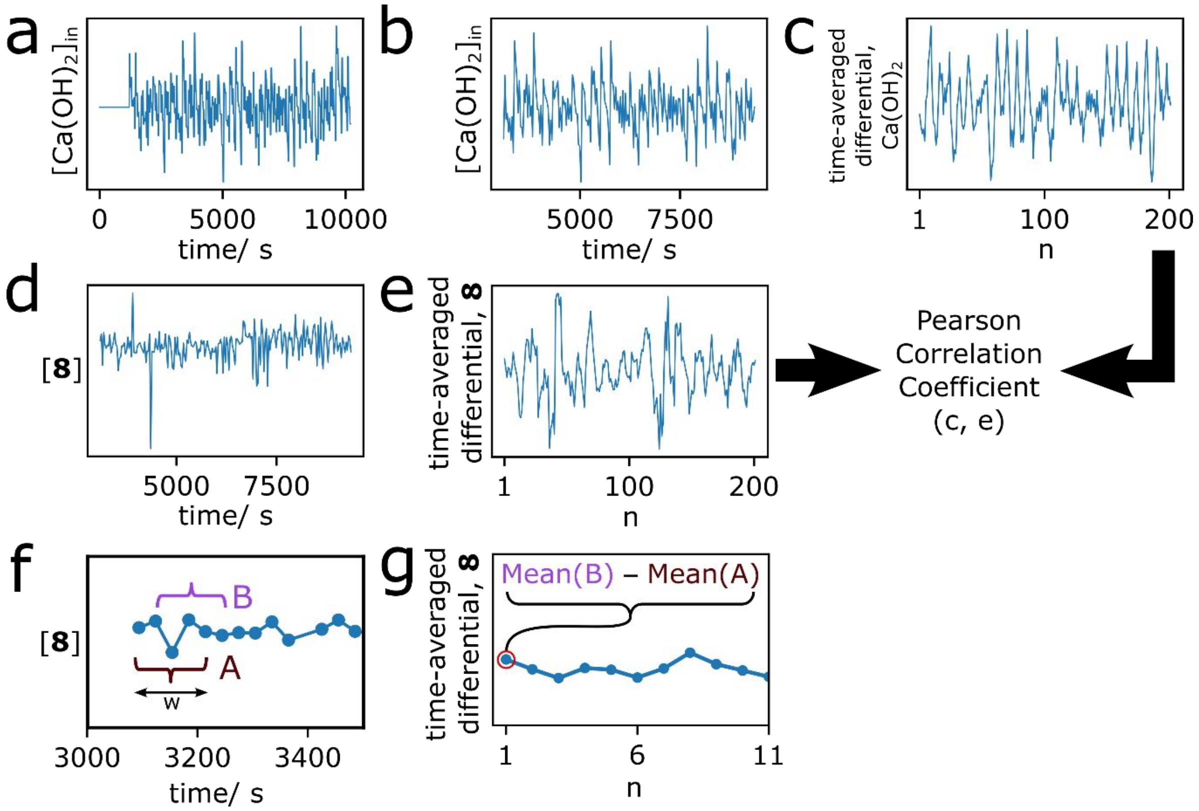

Figure S14. Illustration of the calculation of the time-averaged correlation of a compound (**8**) time course to the dynamic input in EXP013 (see Table 1, main text). This process produced one cell of Figure 4**c** (main text). The dynamic input of $Ca(OH)_2$ (**a**) is resampled by linear interpolation (**b**) to share the same time points as the experimentally determined concentration time courses (**d**). A sliding time-window (width, w=1-5 time points , see **f**) is passed across the input (**b**) and concentration time course (**d**). The mean of each window is subtracted from that of the previous window (**g**) to produce a time-average, differentiated signal for the input and concentration time course (**c** and **e**, respectively). The Pearson correlation coefficient is calculated between the input (**c**) and concentration (**e**) traces to yield a value which can be mapped to the grid in Figure 4**c**.

Supplementary tables

Table S1. Constants for fitted quadratic GC calibration curves for C_4, C_5 and C_6 compounds.

Compound	A	B
DHA, 3	0.028749	0.118714
threose, 5	0.143626	0.789532
erythrose, 6	-0.38468	2.13775
erythrulose, 7	0.143519	0.222204
ribose, 9	0.339712	1.487932
xylose, 10	0.048971	2.23855
xylulose, 11	0.695714	-0.17935
fructose, 17	0.589499	2.049516
sorbose, 18	0.08	2.354559
tagatose, 19	-0.10622	1.017795
8	0.071759	0.111102
12 to 16	0.229793	0.618952
21 to 31	0.250408	1.677321

The same calibration curves have been used as previously reported[1], based on: $[compound] = A \times (\frac{peak\ integral}{internal\ standard\ integral})^2 + B \times \frac{peak\ integral}{internal\ standard\ integral}$. We estimated a calibration curve for non-calibrated compounds (8, 12 – 16, 21 – 31) by taking the average calibration curve for the calibrated sugars of similar length.

References

1. Robinson, W. E., Daines, E., van Duppen, P., de Jong, T. & Huck, W. T. S. Environmental conditions drive self-organization of reaction pathways in a prebiotic reaction network. *Nat. Chem.* **14**, 623–631 (2022).

2. Harris, C. R. *et al.* Array programming with NumPy. *Nature* **585**, 357–362 (2020).
